# 

**Published:** 1881-06

**Authors:** 


### INDEX TO VOLUME XLII.

**A.**

PAGE.

Abscess, alveolar, by E. S. Talbot, M.D ........ 23
Abscess of liver, acute, recovery, S. J. Holmes ........ 273
Address of President of Chicago Gynæcological Society, Wm. H. Byford ........ 13
Address, inaugural, Mary Putnam Jacobi ........ 561
Allport, W. W., response to toast, "Dentistry and Dental Education" ........ 361
Amblyopia from disuse ........ 654
American Graduates in Medicine ........ 559
Analgesia, surgical, Dr. Bossis. 206
Anæsthetic, chloral as an, for children ........ 208
Aneurism—aortic, B. C. Gudden, 491
Ante-partum hæmorrhage—case of spontaneous, G. Frank Lydston ........ 357

**B.**

Beaconsfield and his physicians, J. I. Tucker ........ 487
Belfield, W. T., Foreign Correspondence, Vienna Letter...... 55
Books and Pamphlets Received, 67, 168, 303, 530
Byford, Wm. H., Address of President of the Chicago Gynæcological Society ........ 13

**C.**

Cancer of the breast, case of, recovery after removal, by W. H. Fox ........ 148
Carbolic acid, action of upon ciliated cells and white blood cells ........ 203
Catarrhal diathesis in young girls ........ 658
Cautery, use of actual, in ulceration of cornea, Dr. Fuchs, Vienna ........ 68
Census Bulletin ........ 334

PAGE.

Cervical portion of the sympathetic, experiments on the, M. Gasselein ........ 200
Chemistry, physiological, fowl cholera ........ 194
Chicago Biological Society.... 498
Chicago Medical College, Commencement ........ 553
Chicago Medical Society...500, 508
Children, nocturnal terrors in.. 208
Chloral as an anæsthetic for children ........ 208
Chloral hydrate, by H. H. Kane 35
Chloral hydrate in tetanus, H. H. Kane ........ 242
Chloroform, three deaths from. 187
Clinics...112, 224, 333, 448, 560, 672
Congenital displacement of the lens ........ 652
Cook County Hospital........ 494
Cornea, ulceration of, use of actual cautery in, Dr. Fuchs, Vienna ........ 68
Correspondence, Foreign:
- Vienna letter, W. T. Belfield, 55
- Vienna letter, W. T, Belfield. 290
- Letter from China, Kate C. Bushnell ........ 513
- Vienna letter ........ 636

Correspondence, Domestic:
- Philadelphia letter ........ 58
- New York letter ........
- Boston letter ........ 149
- Letter from Greenwood, Wis.. 157
- Letter from Bloomington, Ill. 158
- New York letter ........ 281

Correspondenc, Domestic:
- Iowa letter ........ 375
- Boston letter ........ 521
- New York letter ........ 638

Cotton pellet as an artificial drumhead, Dr. H. Knapp..... 665
Cystitis, lactic acid in chronic.. 207

**D.**

Deadwood (Dakota), Medical Society, list of members..... 111

PAGE.

Davis, Dr. N. S., examination of the sick........ ........ 1
Davis, Dr. N. S., clinical lecture on erysipelas and diphtheria. 234
Delirium Tremens and acute delirium, treatment of, Dr. Rosseau........ .......... 330
Diphtheria, salt as a prophylactic in........ .......... 223
Diphtheria and erysipelas, clinical lecture by Dr. N. S. Davis, 234
Diphtheria, some points in, C. J. Lewis.................. 347
Diphtheria, the bacterium of, Ch. Talamon, translation..... 397
Diphtheria, some points in, A. T. Conley........ ......... 622
Displacement, uterine, curability of, P. O'Connell....... 128
Dyspepsia, gouty, Germain Sée, translation ................. 510

E.

Editorial:
American Medical College Association.................. 69
Medical legislation, adulteration of food, curiosities in medical teaching, changes in medical journals............ 170
W. W. Allport on dental education....................... 392
Editorial ..... ............... 644
Editorials, condensed from the medical press.............. 532
Education, a review of the progress of medical, in Chicago, E. Ingals.................. 136
Epileptic, mental state of the... 218
Epilepsy and crime........... 374
Errata .................... .. 278
Erysipelas and diphtheria, clinical lecture by N. S. Davis.... 234
Ethyl bromide, internal administration of.................. 51
Etiology of phthisis, Dr. Ed. Labbé...................... 436
Examination of the sick, by N. S. Davis .. ................. 1

F.

Face, the human, modifications in health and disease, and value as guide in diagnosis, A. L. Ranney ............... 77
Foreign substance in trachea... 187
Fox, Wm. H., case of cancer of the breast, recovery after removal ................ ..... 148

PAGE.

G.

Gardiner, E. J., a case of iritis serosa................ ..... 133
Gross, Dr. S. D., clinical lecture, 230
Gynæcological Society of Chicago, address of the president, Wm. H. Byford............ 13

H.

Hæmorrhage, case of spontaneous ante-partum, G. Frank Lydston....... ............ 357
Hip joint disease, a point in treatment of, Norris H. Chapman...................... 359
Homatropine in ophthalmic practice, F. C. Hotz......... 122
Hotz, F. C., homatropine in ophthalmic practice..... ... 122
Hyde, J. Nevins, pityriasis rubra...................... 113
Hydrophobia ...... .. ..... 213
Hydrobromate of morphia..... 391
Hydrorrhœa Gravederum...... 446

I.

Illinois State Medical Society.. 552
Index to Vol. XLI............ 114
Ingals, E., review of progress of medical education in Chicago, suggestions for its advancement .................... 136
Injurious effects of vulcanized rubber, by L. O. Haskell..... 29
Iritis serosa, a case of, E. J. Gardiner................ ..... 133

J.

Jacobi, Dr. A., clinical lecture by ........................ 225
Jacobi, Mary Putnam, inaugural address................. 561

K.

Kane, H. H., chloral hydrate... 35
Kane, Dr. H. H., chloral hydrate in tetanus.................. 242
Keith, Thomas, ovariotomy, Dr. J. Marion Sims............. 319

L.

Lactic acid in chronic cystitis... 207
Lecture, clinical, by Dr. A. Jacobi...................... 225
Lecture, clinical, by Dr. S. D. Gross .......... ......... 230
Lydston, G. Frank, œdema glottitis, difficult laryngotomy... 47

M.

Maternal impression, wonderful case, if true................ 559

PAGE.

Mental state of the persecuted, translated by H. D. Valin... 188
Mercy hospital, surgery in... . 52
Metastasis of mumps.......... 279
Michigan State Board of Health, report of meeting........... 159
Mortality Report.............. 444
Myopia, statistics of, Dr. O. Just, 654

N.

Nasal catarrh, B. T. Mouser.... 430
Nervous diseases, migraine, Dr. Thos. Lathrop............... 305
Nicitating, membrane in man.. 241
Nocturnal terrors in children.. 208
Notice, special................ 304

O.

Obituary, M. O. Heydock, Isaac Ray, Geo. Alexander Otis.... 549
Œdema Glottidis, cellulitis of neck, accompanied by, G. F. Lydston.................... 47
Œsophagism ................ 447
Ophthalmological and otogical literature, abstract of......... 652
Ovariotomy, Thos. Keith and Dr. J. Marion Sims.......... 319

P.

Paralysis, three peculiar cases of, H. D. Valin............ 267
Pathognomonic sign of fracture of neck of the femur.... ... 525
Pityriasis Rubra, J. Nevins Hyde...................... 113
Phosphate of Bismuth, Therapeutic use of............... 332
Physician's leisure, a plea for the study of archæology, Dr. Cheevers.................. 542
Physician's leisure, a plea for the study of archæology, Dr. Cheevers. ................ 660
Physiological chemistry, fowl cholera.................... 194
Phytolacca decandra, W. C. Westerfield .............. 353
Pilocarpine, its effects on syphilis........................ 445
Pregnancy, duration of normal human, P. O'Connell, M.D.... 337
Progress of medical education in Chicago, review of, suggestions for its advancement, E. Ingals ..................... 136
Progress of medicine in the Western States............ 428
Prophylactic, salt as a, in diphtheria.... ................ 223

PAGE.

Prostate, surgical treatment of hypertrophied............... 207
Prussic acid in tobacco smoke.. 217
Psychiatry, a physician's problems in..................... 316
Public health, quarantine...... 75
Pyæmia following mastoid abscess....................... 655
Pygopagi, the.... .... ....... 291
Pyrosis or water brash, treatment of.................... 557

Q.

Quarantine and public health.. 75
Quarantine in diphtheria ..... 429
Quinine amaurosis............. 653

R.

Ranney, A. L., the human face, modifications in health and disease, value as a guide in diagnosis ..... .. .......... 77
Reports of societies:
West Chicago Medical...... 292
Michigan State Board of Health..................... 159
Chicago Medical Society..... 371
West Chicago Medical Society.................. ....... 372
Chicago Biological Society .. 626
Resection of small intestine, recovery.................. 434
Reviews and Book Notices..... 63, 163, 294, 385, 526, 644
Rubber, injurious effects of vulcanized, by L. P. Haskell.... 29
Rush Medical College... ..... 439

S.

Sanitary Convention .... ..... 280
Scotch introductory addresses, S. D. Campbell Black......... 407
Selections.................... 402
Sick, examination of the, by N. S. Davis.................... 1
Skene, J. C., diseases of the glands of the female urthra.. 177
Society meetings............... 112, 224, 333, 448, 560, 672
Society Reports:
Chicago Medical Society.... 630
West Chicago Medical Society......................... 634
Spinal Bifida, treated with plaster of Paris................. 384
State Board of Health of Illinois......................... 378
State Board of Health of Michigan....................... 504
Statistics of medical journals.. 558

PAGE.

Stretching the facial nerve for relief of spasms of facial muscles .... 313
Suppositories, beef .... 54
Surgery in Mercy Hospital..... 52
Surgical analgesia, Dr. Bossis.. 206
Sweating, morbid .... 210

T.

Tetanus, chloral hydrate in, Dr. H. H. Kane .... 242
Tetrachloride of carbon, aromatic .... 393
Texas, mountain region of Western, as a health resort, F. C. Lawrence .... 287
The thermograph, its evolution and destiny, A. Wellington Adams .... 412
Therapeutics, advance in, during 1880 .... 370
Trachea, foreign substance in.. 187

PAGE.

Translations, latest experiments with virus of hydrophobia, by Bouley and Pasteur...... 394

U.

Urethra, diseases of the glands of the female, J. C. Skene.... 177
Uterine displacement, curability of, P. O'Connell .... 128

V.

Vaccine virus, O. C. DeWolf... 481
Venereal outrages, simulations of, on young children........ 221

W.

Whooping cough, treatment of by inhalations of spirits of turpentine .... 222
Woman's Medical College..... 293

Y.

Yellow fever, H. D. Schmidt... 449, 585

SOCIETY MEETINGS.

Chicago Medical Society—Mondays, June 6-20.

West Chicago Medical Society—Mondays, June 13-27.

Biological Society—Wednesday, June 1.

CLINICS.

MONDAY.

Eye and Ear Infirmary—2 p. m., Ophthalmological, by Prof. Holmes; 3 p. m., Otological, by Prof. Jones.

Mercy Hospital—2 p. m., Surgical, by Prof. Andrews.

Rush Medical College—2 p. m., Dermatological and Venereal, by Prof. Hyde.

Woman's Medical College—2 p. m., Dermatological and Venereal, by Prof. Maynard; 3 p. m., Diseases of the Chest, Prof. Ingals.

TUESDAY.

Cook County Hospital—2 to 4 p. m., Medical and Surgical Clinics.

Mercy Hospital—2 p. m., Medical, by Prof. Quine.

WEDNESDAY.

Chicago Medical College—2 p. m., Eye and Ear, by Prof. Jones.

Rush Medical College—2 p. m., Medical, by Dr. Bridge; 3 p. m., Ophthalmological and Otological, by Prof. Holmes; 3:30 to 4:30 p. m., Diseases of the Chest, by Dr. E. Fletcher Ingals.

THURSDAY.

Chicago Medical College—2 p. m., Gynæcological, by Prof. Jenks.

Rush Medical College—2 p. m., Diseases of Children, by Dr. Knox; 3 p. m., Diseases of the Nervous System, by Prof. Lyman.

Eye and Ear Infirmary—2 p. m., Ophthalmological, by Dr. Hotz.

Woman's Medical College—3 p. m., Surgical, by Prof. Owens.

FRIDAY.

Cook County Hospital—2 to 4 p. m., Medical and Surgical Clinics.

Mercy Hospital—2 p. m., Medical, by Prof. Davis.

SATURDAY.

Rush Medical College—2 p. m., Surgical, by Prof. Gunn; 3 p. m., Orthopœdic, by Prof. Owens.

Chicago Medical College—2 p. m., Surgical, by Prof. Isham; 3 p. m., Neurological, by Prof. Jewell.

Woman's Medical College—11 a. m., Ophthalmological, by Prof. Montgomery; 2 p. m., Gynæcological, by Prof. Fitch.

Daily Clinics, from 2 to 4 p. m., at the Central Free Dispensary, and at the South Side Dispensary.

### INDEX TO PROCEEDINGS.

PAGE.

FIRST DAY ........ 673
President's Address ........ 674
Report of the Foreign Delegates ........ 681
SECOND DAY ........ 682
Special Order—Amendment to Code of Ethics ........ 682
Report on Necrology ........ 683
Address of Dr. Wm. Pepper, Chairman of Section on *Practical Medicine* ........ 684
Address of Dr. J. R. Chadwick, Chairman of Section on *Obstetrics* and *Diseases of Women and Children* ........ 697
THIRD DAY ........ 703
Address of Dr. Hunter McGuire, Chairman of Section on *Surgery*, on *Operative Interference in Gunshot Wounds of Peritoneum* ... 703
Paper by Dr. J. S. Billings, U. S. A., on *Some of the Results of the Tenth Census as Regards Mortality Statistics* ........ 707
Report by Dr. N. S. Davis, on *Clinical and Meteorological Records*. 711
FOURTH DAY ........ 712
Amendment to Code of Ethics ........ 713
Report of Committee on Nominations ........ 713
Report of Committee on Official Position of Members of the Medical Staff of the Army and Navy ........ 714
Resolutions of Thanks ........ 714

### PROCEEDINGS OF SECTIONS.

*Section on Practice of Medicine, Materia Medica and Physiology:*
First Day.—Blood-letting as a Therapeutic Measure in Pneumonia. By Dr. W. C. Wile ........ 715
Second Day.—Is Croupous Pneumonia a Zymotic Disease? By Dr. Prentiss ........ 721
Nature and Treatment of Pneumonia. By Dr. W. C. Dabney ... 721
Third Day.—The Diet and Hygiene of Eczema. By Dr. L. Duncan Bulkley ........ 724
Treatment of Diphtheria. By Dr. Whittaker ........ 726
On the Production of Albuminuria by Use of Iodide of Potassium in Syphilitic Disease. By Dr. I. E. Atkinson ........ 727
Variola Vacciniæ and Variola Equiniæ in Massachusetts. By Dr. Henry A. Martin ........ 728
The Materia Medica of the Future. By Dr. F. E. Stewart ...... 728

*Section on Obstetrics and Diseases of Women:* PAGE.
First Day.—Résumé of Rules for the use of Pessaries. By Dr. Paul F. Mundé .......... 729
Second Day.—Investing Capsule of Uterine Fibroids. By Dr. F. C. Larrimore .......... 734
Exhibit of the Uterine Dilators. By Dr. H. P. C. Wilson...... 735
Third Day.—Can we Make a Positive Diagnosis of Pregnancy Previous to the Occurrence of the Audible Sounds of the Fœtal Heart and the Detection of the Fœtal Movements? By Dr. Joseph Tabor Johnson .......... 737

*Section on Surgery and Anatomy:*
First Day.—Exhibit of a Collection of Instruments. By Dr. Joseph H. Warren .......... 741
Report of a Case of Pyonephrosis. By Dr. B. W. Allen........ 742
Second Day.—A New System of Surgical Mechanics. By Dr. Charles F. Stillman .......... 743
Plastic Operations on the Face. By Dr. Alfred C. Post........ 744
Treatment of Arthritis of the Temporo-Maxillary Articulation. By Dr. D. H. Goodwillie .......... 745
Third Day.—An Experimental and Clinical Inquiry into the Etiology and Distinctive Peculiarities of Traumatic Fever. By Dr. B. A. Watson .......... 747
Labial Carbuncle or Malignant Pustule of the Lip. By Dr. Charles A. Leale .......... 750

*Section on State Medicine:*
Second Day.—The National Board of Health and the International Sanitary Conference of 1881. By Dr. J. L. Cabell............ 753
On the Relation of the State to the Insane. By Dr. C. F. Folsom. 753
Cellars. By Dr. A. N. Bell .......... 754
The Necessity and Means of Removing Putrescible Matters from Inhabited Places. By Dr. O. W. Wright .......... 754

*Section on Ophthalmology, Otology and Laryngology:*
First Day.—On the Perimeter. By Dr. G. T. Stephens........... 754
Nasal Catarrh with Hypertrophy. By Dr. W. C. Jarvis........ 755
Treatment of Conical Cornea. By Dr. Chisholm.............. 758
Second Day.—Syphilitic Laryngitis. By Dr. Carl Seiler......... 758
A Form of Tinnitus Induced by a Rhythmical Contraction of the Tensor Tympani Muscle. By Dr. Chisholm .......... 760

*Section on Diseases of Children:*
First Day.—The Relation between Growth and Disease. By Dr. H. I. Bowditch .......... 763
Some of the Causes of Infantile Eczema, and the Importance of Mechanical Restraint in its Treatment. By Dr. White....... 764
Second Day.—Treatment of Diphtheria. By Dr. R. J. Nunn..... 770
Third Day.—Middle-Ear Disease in Children in the Course of the Acute Exanthemata. By Dr. Blake .......... 774

### Supplement to the June Number of the

### Chicago Medical Journal & Examiner

VOLUME OF 1881.

PROCEEDINGS OF THE

### AMERICAN MEDICAL ASSOCIATION,

AT THE

THIRTY-SECOND ANNUAL MEETING,

HELD IN

RICHMOND, VIRGINIA, MAY 3, 4, 5 AND 6, 1881.

CHICAGO
PUBLISHED BY THE CHICAGO MEDICAL PRESS ASSOCIATION,
188 CLARK STREET.

### SPECIAL NOTICE.

We present to our readers with this number of the Chicago Medical Journal and Examiner, a supplement of 112 pages, in which will be found a full report of the proceedings of the late meeting of the American Medical Association, collated in part from the daily edition of the *Virginia Medical Monthly*, the Editors of which deserve much credit for their efforts to publish daily full reports. This large addition to the cost of the Journal for the year and to the number of pages in the annual volume, is gained without any increase in the price of the Journal to our subscribers. The report, it is believed, is more full than any which has yet appeared, and we trust that this enterprise on the part of the Editors and Publishers will receive the appreciation which it is aimed to secure. We beg leave to remind our friends at this time, also, that we expect soon to present them with an excellent report of the transactions of the International Medical Congress, and that in our next issue we expect to report the proceedings of the Illinois State Medical Society, which is now in session in this city. No pains nor expense will be spared, in the future as in the past, to keep this Journal of Medicine in the forefront of all enterprises having in view the advancement of the interests of the profession.

